# Western Diet Induces Changes in Gene Expression in Multiple Tissues During Early Insulin Resistance and Glucose Intolerance in Male C57BL/6 Mice

**DOI:** 10.3390/cimb47121053

**Published:** 2025-12-16

**Authors:** Djordje Radulović, Jurij Dolenšek, Andraž Stožer, Uroš Potočnik

**Affiliations:** 1 Center for Human Genetics and Pharmacogenomics, Faculty of Medicine, University of Maribor, Taborska Ulica 8, 2000 Maribor, Slovenia; djordje.radulovic@student.um.si; 2 Institute of Physiology, Faculty of Medicine, University of Maribor, Taborska Ulica 8, 2000 Maribor, Slovenia; jurij.dolensek@um.si; 3 Faculty of Natural Sciences and Mathematics, University of Maribor, Koroška Cesta 160, 2000 Maribor, Slovenia; 4 Laboratory for Biochemistry, Molecular Biology and Genomics, Faculty of Chemistry and Chemical Engineering, University of Maribor, Smetanova Ulica 17, 2345 Maribor, Slovenia; 5 Department for Science and Research, University Medical Centre Maribor, Ljubljanska Ulica 5, 2000 Maribor, Slovenia

**Keywords:** dedifferentiation, gene expression, glucose-intolerant mice, *PPARγ*, *Tcf7l2*, type 2 diabetes, Western diet

## Abstract

To better understand the molecular mechanisms by which a Western diet (WD) promotes the development of type 2 diabetes (T2D), we analyzed changes in the expression profiles of multiple glucose-regulatory tissues of male C57BL/6 mice. We fed the mice with either a regular control diet (CD) or a WD. Standard glucose and insulin tolerance tests were performed, and body weight, plasma glucose, and triglyceride levels were measured to assess the glucose homeostasis *in vivo*. The WD induced obesity, glucose intolerance, and insulin resistance, with a fasting hyperglycemia. Further, we identified several changes in the gene expression of the analyzed candidate genes in all the examined target tissues, including the downregulation of *Tcf7l2* in the liver, pancreas, white and brown adipose tissue (0.72, 0.56, 0.36, and 0.22-fold, respectively), in contrast to a marked upregulation in the intestine (2.57-fold). We also found downregulation of *PPARγ* in the white and brown adipose tissue (0.55, 0.41-fold, respectively) and upregulation in the pancreas, liver, intestine, and skeletal muscle (1.25, 1.46, 1.97, and 2.59-fold, respectively). Our findings provide important insight into the characteristic pattern of changes in expression of candidate genes during the early stages of insulin resistance and glucose intolerance in this diet-induced mouse model of T2D.

## 1. Introduction

Type 2 diabetes (T2D) is a widespread lifestyle metabolic disorder and one of the leading causes of mortality across the globe. It is characterized by decreased insulin sensitivity (IS), also termed increased insulin resistance (IR), initial beta cell compensation with hyperinsulinemia, and eventual beta cell dysfunction and progression to glucose intolerance and hyperglycemia [1]. Plant-based dietary patterns reduce the risk of developing IR in both humans and experimental animals [2,3,4]. In contrast, a diet rich in fat, refined grains, and sucrose, which is typical for a Western dietary pattern (WD), is associated with an increased risk of developing obesity, IR, hyperglycemia, and T2D [5,6,7]. However, the relationship between diets of this type and the dysregulation of insulin sensitivity in target tissues and beta cell dysfunction during the onset and progression of T2D remains controversial. Strictly controlled processes maintain glucose homeostasis in mammalian cells and represent an important and evolutionarily conserved survival mechanism [8]. Postprandially, insulin suppresses hepatic gluconeogenesis and promotes the uptake and storage of excess glucose and other energy-rich nutrients by the liver and by the peripheral tissues simultaneously, such as adipose tissue and skeletal muscle, to enable storage of excess energy and its utilization between meals. However, significant metabolic abnormalities occur in the diabetic state. The liver typically diminishes glycogen synthesis and displays enhanced gluconeogenesis [9]. On the other hand, the increased glucose uptake in the intestinal enterocytes probably also contributes to hyperglycemia [10] and likely plays a key role in the development of obesity and T2D [11]. According to previous findings, promising innovative strategies in dealing with obesity and improvement in insulin sensitivity can be based on energy expenditure by activating brown adipose tissue (BAT) [12,13]. However, despite the fact that BAT is considered as T2D protective tissue, disturbance in the functions of this specific type of adipose tissue may also contribute to the development of T2D [14]. One of the most important mechanisms of insulin action is the inhibition of lipolysis in white adipose tissue (WAT), promotion of proper adipocyte differentiation, lipogenesis, fat storage, glucose intake, and regulation of adipokine production and secretion [15]. However, under conditions of energy overload, this important role of insulin can be disrupted, and manifested by an increase in the circulating free fatty acids (FFAs), with a consequent accumulation of lipids in non-adipose tissues. In the skeletal muscles, FFAs are known to inhibit insulin-mediated glucose uptake, thereby reducing glycogen production and increasing intramyocellular lipid levels, which contribute further to the development of skeletal muscle IR [16,17,18,19]. Of significance, insulin-resistant skeletal muscles are characterized by a decreased ability for postprandial glucose uptake, and thus are implicated indirectly in the compensatory increased glucose uptake by the liver, where hepatic de novo lipogenesis is stimulated and associated with the development of hepatic steatosis [20]. In addition, another strong factor implicated in the promotion of lipid accumulation and liver dysfunction, and, thus, T2D progression, is disturbed intestinal function under conditions of overnutrition [21]. Also, ectopic accumulation of FFAs in the islets of Langerhans is associated with the development of disorders of insulin secretion, beta cell stress and dysfunction, which, in turn, worsens the glucose intolerance and T2D [22]. Remarkably, studies have shown that chronic exposure of beta cells to increased levels of glucose and lipids is associated with a loss of fully differentiated beta cells [23].

To gain further insight into the mechanistic changes in the pancreas and target tissues at the level of expression changes in key candidate genes, we focused on a few selected genes that have been implicated in T2D pathogenesis. This study investigates how a 12-week Western diet (WD) consumption alters the expression of key T2D-related genes (*Ins2*, *Glut2*, *Pdx1*, *MafA*, *Nkx2.2*, *MafB*, *GcG*, *Sox9*, *C-Myc*, *Ngn3*, *PPARγ*, *Hnf-1α*, *Lrp5*, and *Tcf7l2*) across major metabolic tissues in male C57BL/6J mice, and the selection of these genes is justified in the following section.

The selected genes play key tissue-specific regulatory roles, coordinating multiple metabolic processes, including glucose homeostasis, insulin sensitivity, and lipid metabolism [24,25,26,27,28,29,30,31,32,33]. Together, this is relevant for studying the molecular mechanisms associated with metabolic disorders such as insulin resistance, obesity, and T2D. The targeted *Tcf7l2*, *Hnf-1α*, and *PPARγ* genes play a fundamental role in regulating evolutionarily conserved signaling and transcriptional pathways in metabolically important tissues, thereby maintaining the functionality and homeostasis of target organs and tissues [34,35,36]. *Lrp5* was selected as a key component of the Wnt/β-catenin signal pathway [27] and, as such, plays a central role in maintaining the functionality of both white and brown adipose tissue [37]. Further, *Glut2* [38] was examined in the intestine, liver, and pancreas to assess tissue-specific glucose transport and adaptive responses to nutritional overload. In the pancreas, specific markers of adult endocrine beta and alpha cells were analyzed to gain further insight into their functionality, as well as progenitor *Ngn3*, *C-Myc*, and *Sox9* genes [39,40,41] to assess dedifferentiation, i.e., whether changes in the identity of mature adult beta cells occur, and the potential neogenesis of pancreatic endocrine cell lineages under metabolic stress. This panel provides a comprehensive view of specific molecular changes induced by a WD across multiple glucose-regulatory tissues simultaneously.

Of interest, Schmitt et al. demonstrated that targeted deletion of *Glut2* (also known as *Slc2a2*) encoding the facilitative hexose transporter GLUT2 specifically in the mouse intestine restricts intestinal permeability, sugar absorption, and limits weight gain [42]. These data suggest that the increase in the *Glut2* gene expression has a negative in vivo impact on glucose homeostasis in mice. Moreover, GLUT2 is the main isoform in the hepatocytes and rodent islet beta cells [38,43,44,45]. Previously, it has been reported that *Glut2* gene expression is upregulated in the intestine/liver and downregulated in the pancreatic tissue of diabetic animals and humans [38,46,47,48,49]. Thus, disturbance in the *Glut2* expression is strongly associated with the pathogenesis of T2D.

The transcription factor HNF-1α (hepatocyte nuclear factor 1α) is expressed highly in the liver, but also in the pancreas, digestive tract, and kidneys at lower expression levels, and plays an important role in regulating the activity of the numerous genes involved in lipid and glucose metabolism and transport in the intestine and liver [24,25]. The experimental data obtained by Servitja et al. suggest that HNF-1α may be a major regulator of beta cell growth [35], and it is essential for the pancreatic islet development, metabolism, regulation of *Glut2* expression, and other key transcription factors which are involved in the pancreatic beta cell differentiation [50,51,52]. However, Luco et al. found that HNF-1α overexpression in the pancreatic beta cells has a deleterious effect on the expression of beta cell-enriched and specific genes, such as *Glut2* and *Pdx1*, as well as beta cell proliferation and function [53].

The Wnt/β-catenin signaling is associated with obesity, hyperlipidemia, hyperglycemia, islet function, and T2D [26,27,28]. In addition, both aberrant Wnt/β-catenin signaling and hyperglycemia are associated with an increased risk of cancer in multiple organs, including the gastrointestinal tract, in individuals with diabetes [54,55]. Genome-wide association studies (GWAS) have revealed that the gene of the Wnt signaling pathway *Tcf7l2* that encodes T cell-specific transcription factor 7-like 2 (TCF7L2) is the most important human T2D risk factor discovered to date [56]. Previously, it has been demonstrated that TCF7L2 has a beneficial effect on the expression of the key islet beta cell transcription factors (MAFA, PDX-1, NKX6.1), and that the activation of the β-catenin/TCF7L2 signaling mechanism promotes new beta cell formation, survival, and regeneration [57]. Previous work indicated that the *GcG* gene in intestinal endocrine L cells, which encodes for the incretin hormone products such as GLP-1 (glucagon-like peptide-1), essential for the maintenance of glucose homeostasis and normal beta cell functions, is among the targets of the Wnt/β-catenin signaling pathway [58]. Insulinotropic GLP-1 and diabetogenic glucagon hormone, encoded by *GcG*, have opposite effects on glucose homeostasis [59,60,61]. Columbus et al. found that insulin treatment and high-fat diet (HFD) feeding reduce the expression of *Tcf7l2* mRNA in a rodent pancreas, contrary to human and mouse intestinal cell lines, where insulin stimulated both *Tcf7l2* and *GcG* mRNA expression [62]. Besides the regulation of the hormonal function in intestinal endocrine L cells, there is accumulating evidence that the downstream Wnt/β-catenin signaling effector encoded by *Tcf7l2* is an important regulator of hepatic gluconeogenesis [34]. Furthermore, the Wnt/β-catenin and the insulin signaling pathway have essential but opposite roles in maintaining normal adipose tissue biology [15,27,63,64,65]. Previously, it has been reported that *Tcf7l2* expression is reduced in human insulin-resistant subcutaneous adipose tissue [66]. In addition, the Wnt/β-catenin signaling pathway co-receptor LRP5 has also been associated with obesity and T2D [67,68,69,70]. Altogether, these findings highlight that the disturbance in the expression of genes encoded for the up- and downstream Wnt/β-catenin signaling pathway components TCF7L2 and LRP5 has an important and significant role in the pathogenesis of T2D. However, it is important to emphasize that, to the best of our knowledge, none of the studies to date have examined simultaneously *Tcf7l2* expression levels in all the most important target tissues (intestine, adipose tissue, liver, skeletal muscle, and pancreas) during the progression to obesity and diabetes induced by a 12-week WD.

Both central visceral adiposity and overweight are very serious public health issues, since they increase the risk for metabolic diseases and T2D significantly. The ability of white adipocytes to take up and store accessible triglycerides depends on the relative size of the fat depots and is regulated strictly through the well-established mechanisms [71,72]. However, it is known that overnutrition leads to disturbances in adipose tissue function [73]. Loh et al. demonstrated that the Wnt/β-catenin signal pathway via LRP5 signaling regulates both adipose progenitor biology and regional adiposity, thereby modulating hyperplasia and hypertrophy, and, thus, fat distribution and fat depot capacity [69].

PPARγ has a crucial role in adipose tissue development and has strong antidiabetic effects through its ability to promote lipid storage and thermogenesis. Unfortunately, the influence of a 12-week WD on the WAT/BAT functionality is underestimated, and data related to adipocyte dysfunction of both types are very limited. Although *PPARγ* was recognized as an adipocyte master gene, the regulation of *PPARγ* expression and the activity of its protein product are also crucial for the function and homeostasis of other organs and cells in the body, not only adipocytes and adipose tissue. In contrast to adipose tissue, *PPARγ* is less expressed in the skeletal muscle, pancreas, small intestine, and hepatocytes in adult rats and mice [74,75,76]. Previous studies suggest that the aberrant *PPARγ* activation may be associated with an adipogenic redirection of satellite cells [77,78,79], lipid accumulation in the skeletal muscles [80], and other tissues. It is noteworthy that chronic lipid accumulation in the islets of the Langerhans can exhaust the beta cells and cause a lipotoxic beta cell phenotype, followed by beta cell dysfunction, reduction in insulin content, and increased beta cell apoptosis [22]. However, the mechanisms linking the overall impact of WD to beta cell failure remain largely unknown.

Considering all stated above, the main view of T2D pathogenesis focuses on the underlying mechanisms that are associated with and responsible for the final decline in the pancreatic beta cell function due to increasing hyperglycemia in T2D individuals. Namely, progressive T2D is characterized by significant decreases in the beta cell mass and insulin secretion. The decrease in the functional beta-cell mass associated with T2D has been attributed primarily to beta-cell death. However, increasing evidence suggests that phenomena such as beta cell dedifferentiation and dysfunction are an important possible alternative mechanism of loss of adult functional beta cell mass in hyperglycemic conditions [81]. Dedifferentiated beta cells represent a less mature form of this specific pancreatic cell type, characterized by reduced expression of the key genes involved in the maintenance of the mature phenotype [82] and re-expression of progenitor genes, such as *Ngn3* and *c-Myc* [39,40]. This novel concept of beta cell failure in T2D is now supported strongly in humans and rodents [40,83,84]. To our knowledge, it is not known whether WD contributes to the development of the diabetic phenotype by inducing beta cell dedifferentiation. Interestingly, pancreatic ductal cells are multipotent progenitors, and the ductal epithelium is believed to be one of the cell sources for differentiation or trans-differentiation into beta cells [85]. Furthermore, SOX9 is essential for the maintenance of pluripotent pancreatic progenitor cells [41]. Importantly, it has been shown that SOX9 is required for *Ngn3* expression, but high levels of activated NGN3 downregulate *Sox9* expression, and, thus, initiate endocrine cell differentiation [86]. On the other hand, the homeodomain transcription factor NKX2.2 is the key regulator of pancreatic islet cell specification, the final differentiation of both insulin and glucagon-producing cells, beta cell maturation, a critical regulator of maintaining proper mature beta cell function, and in forming correct islet architecture [87]. However, the repressor activities of NKX2.2 are sufficient for the differentiation of the alpha cell type in mice [88]. In the conditions of beta cell destruction, O’Reilly et al. demonstrated increased duct cell proliferation, differentiation, and alpha cell neogenesis in the ducts of diabetic NOD mice [89]. In addition to this, Yoon et al. found the presence of glucagon and insulin-positive cells in the pancreatic ducts, and a significant increase in the alpha cell fraction in T2D Korean patients [90]. If we take into account these previous findings, not only beta cell dysfunction, dedifferentiation, and apoptosis, but also aberrant endocrine progenitor cells are a key mechanism in the development of T2D. To date, the precise mechanisms involved in the beta cell deficiency and in the rise in glucagon levels seen in T2D individuals are still not fully understood.

This study aims to clarify unresolved questions about how the WD drives beta cell dedifferentiation, disrupts insulin signaling, and alters tissue-specific gene regulation during T2D progression. By exploring gene-tissue interactions and signaling pathways like Wnt/β-catenin and PPARγ, the study addresses gaps in understanding the mechanisms linking diet to beta cell failure and systemic insulin resistance.

## 2. Materials and Methods

### 2.1. Experimental Animals and Diet

In this study, we used six (*N* = 6), 12-week-old, male C57BL/6 mice, purchased from Charles River (Montreal, QB, Canada). The number of animals used in this study was minimized according to the 3Rs while still preserving statistical power to detect differences during in vivo tests. At the start, the age and body weight-matched experimental mice were randomized into two experimental groups. The mice used in this experiment were maintained in an environmentally controlled room (22–24 °C) under a regular 12 h/12 h light/dark cycle. Prior to starting diet intervention, animals were allowed 2 weeks to adjust to handling during ipGTT and ipITT. The free-feeding animals (*N* = 3 per group) received a normal chow diet (CD; R70, Lantmännen, Stockholm, Sweden) or Western diet (WD; D12079B, Research Diets Inc., New Brunswick, NJ, USA) for 12 weeks with food and water ad libitum. The detailed nutritional profiles of the CD and WD are presented in Table 1 (below) and also available in Supplementary Table S2.

At the age of 24 weeks, we performed an intraperitoneal glucose tolerance test (ipGTT): the mice were fasted overnight, followed by an i.p. injection of a glucose solution (2 g/kg in 0.9% NaCl solution). The plasma glucose concentration was measured using the tail-vein-puncture method with a glucometer (FreeStyle Glucometer, Abbot Diabetes Care, Illinois, USA) immediately prior to the glucose injection (time point 0), and at time points 15, 30, 60, 90, and 120 min after the glucose injection. At the age of 25 weeks, we performed an intraperitoneal insulin tolerance test (ipITT): the mice were fasted overnight, followed by i.p. injection of insulin (0,75 U/kg in 0.9% NaCl solution) at time point −10, and an i.p. injection of glucose (1 g/kg in 0.9% NaCl solution) at time point 0 min. The plasma glucose concentration was measured using the tail-vein-puncture method with a glucometer (FreeStyle Glucometer, Abbot Diabetes Care, Abbott Park, IL, USA) immediately prior to the insulin and prior to the glucose injection, and at the time points 15, 30, 60, 90, and 120 min after the glucose injection. After the ipITT, the mice were euthanized with CO_2_ and cervical dislocation, and we isolated the pancreatic tissue immediately, followed by the duodenum, liver, white adipose tissue, brown adipose tissue, and m. gastrocnemius. The harvested tissue samples were washed twice in ice-cold ribonuclease-free PBS, snap-frozen immediately, and kept in a deep freezer at −80 °C until further RNA extraction and analyses. The animal care and all the experimental procedures were carried out according to the guidelines from the local authorities and the Ethical Committees.

### 2.2. RNA Isolation and Quantitative RT-PCR

The total RNA from the harvested tissue was isolated with a miRNeasy Micro Kit (50) (Qiagen, Hilden, Germany), following the manufacturer’s recommendations. The purity, quantity, and concentration of the extracted RNA were assessed by spectrophotometry (Nanodrop 2000; Thermo Fisher Scientific, Waltham, MA, USA). Only RNA was used with absorbance ratios of approximately 2.0 for 260/280 nm. The RNA integrity was checked by agarose gel electrophoresis and LabOnChip (Agilent Technologies, Santa Clara, CA, USA), and only RNAs with high integrity (RIN > 7) were used for further experiments. The RNA (1 μg) was reverse-transcribed using a High-Capacity cDNA Reverse Transcription kit (Applied Biosystems™ Thermo Fisher Scientific, Waltham, MA, USA). The RT-qPCR was performed with a Fast SYBR green master mix (Applied Biosystems™, Thermo Fisher Scientific, Waltham, MA, USA) using a QuantStudio 12K Flex Real-Time PCR System (ThermoFisher Scientific, Waltham, MA, USA). The comparative Ct (2^−ΔΔCT^) method was used to calculate the relative expression of the candidate genes [91]. The data were normalized using Beta-2-Microglobulin (*β2M*) as an internal control, and calculated fold-change compared to the CD-fed mice. Each sample per tissue from each mouse (six mice total: 3 CD, 3 WD) per experiment was analyzed in triplicate, with negative controls included on all plates. Melting curves were examined to assess the quality of RT-qPCR amplification for each sample, and comparisons between groups were performed using an unpaired *t*-test with Welch’s correction, applied when variances were unequal. The primer sequences are summarized in Supplementary Table S1 [62,92,93,94,95,96,97,98,99,100,101,102,103,104].

### 2.3. Statistical Analysis

**Figure 1:**
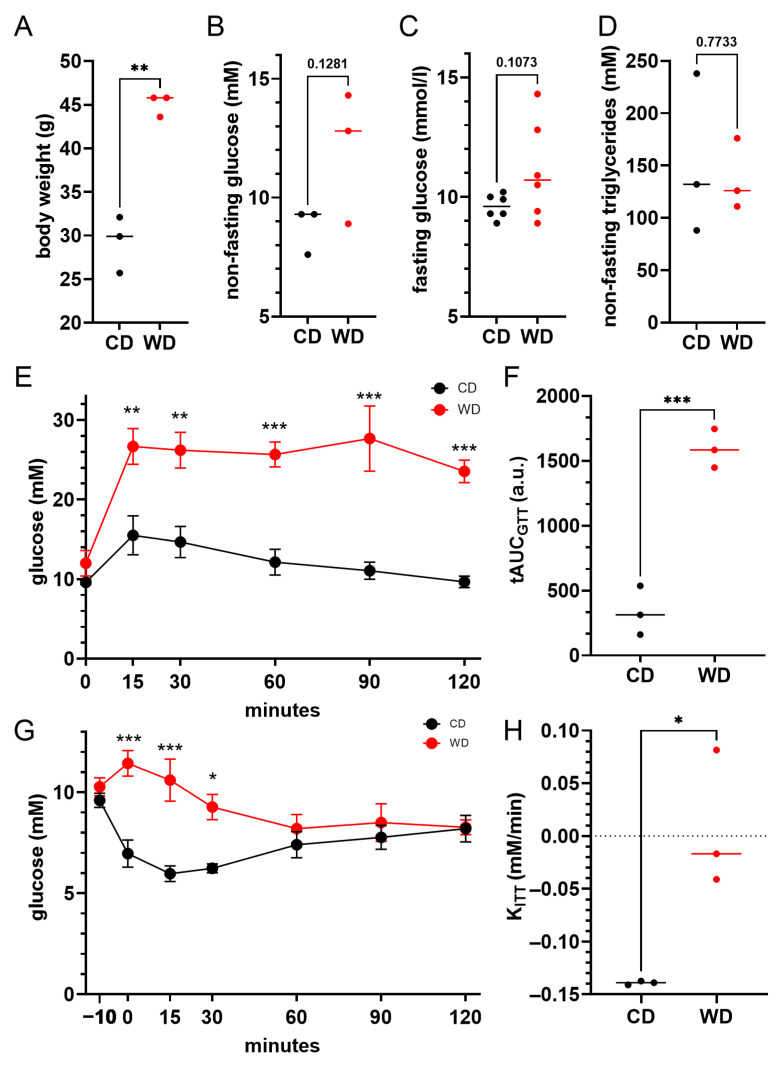
WD-induced diabetes in mice. (**A**–**D**) Body weight (**A**), non-fasting (**B**) and fasting (**C**) plasma levels of glucose, and non-fasting levels of triglycerides (**D**) after 12 weeks of either CD (black) or WD (red) feeding. (**E**) Glucose level excursions after ipGTT in WD- (red) and CD-fed (black) animals. (**F**) Quantification of panel (**E**) by calculation of the total area under the ipGTT curve (tAUC). (**G**) Changes in the glucose levels during ipITT in WD- (red) and CD-fed (black) animals. (**H**) Quantification of panels (**G**) by calculation of the slope of the change in glucose concentration during the first 25 min of the ipITT. Detailed description of data is in the main text. * *p* < 0.05, ** *p* < 0.01, *** *p* < 0.001.

The results for all the measurements are expressed as means with a standard error of the mean (± SEM). The statistical differences between the two experimental groups were determined by an unpaired two-tailed Student’s *t*-test, unless indicated otherwise. *p*-values < 0.05 were considered statistically significant, and are denoted with asterisks (* *p* < 0.05, ** *p* < 0.01, *** *p* < 0.001, **** *p* < 0.0001). A statistical analysis of all the data was performed using GraphPad Prism (version 8.4.3; GraphPad Software, San Diego, CA, USA). The ipGTT and ipITT were analyzed using the two-way ANOVA (Sidak multiple comparisons test) [105], and Figure 1A–D,F–H using *t*-tests. The glucose clearance during ipGTT was quantified further by pooling the total AUC values per mouse. To quantify the response to insulin injection, we calculated the slope of the glucose change during the first 25 min after the insulin injection (K_ITT_) [105].

## 3. Results

The mice fed with the WD were overweight (mean 45.1 vs. 29.2 g, *p* < 0.01, Figure 1A), and exhibited non-fasting (12.0 vs. 8.7 mM, *p* = 0.13, Figure 1B) and fasting hyperglycemia (11.1 vs. 9.6, *p* = 0.11, Figure 1C). The non-fasting levels of plasma triglycerides were comparable between the two groups (Figure 1D). To quantify the glucose handling and glucose clearing in vivo, we performed ipGTT and ipITT tests (Figure 1E–H). The WD-feeding resulted in increased glucose levels during the ipGTT test (Figure 1E), corroborated by a more than threefold difference in the AUC of the ipGTT curves (337 vs. 1593 a.u., *p* < 0.001). To quantify the response to insulin injection, we calculated the slope of the glucose change during the first 25 min after the insulin injection (K_ITT_). tAUC was omitted in analysis as the time points in the later stages of ipGTT reflect more the counterregulatory action of glucose-increasing hormones rather than insulin [105,106]. The glucose clearance, induced by insulin injection during ipITT, was inhibited greatly (Figure 1G), corroborated by a change in the K_ITT_ from −0.15 mM/min to about zero (*p* < 0.05) during the first 25 min following the insulin injection.

**Figure 2:**
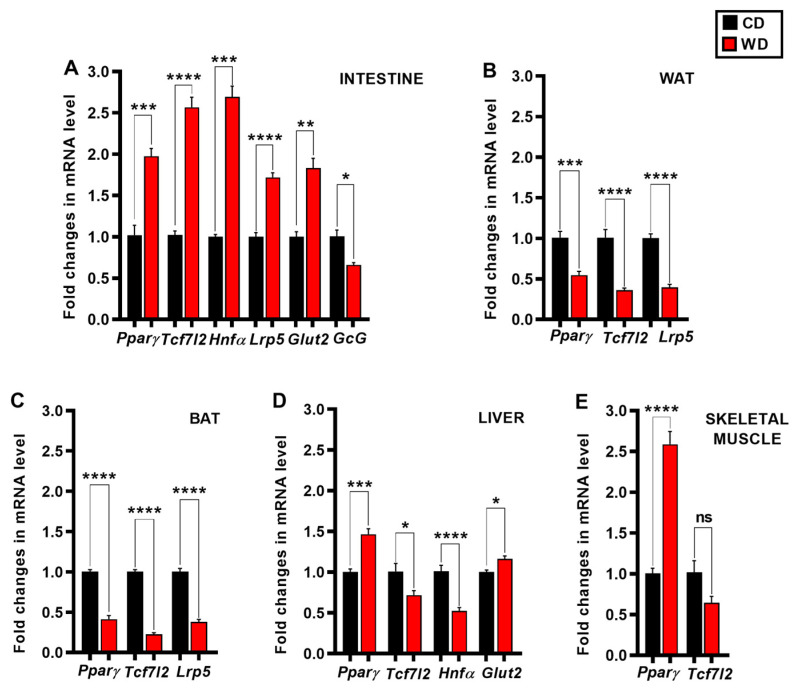
The effect of a 12-week Western diet feeding on the expression levels of the diabetes related genes in the intestine, adipose tissue, liver, and skeletal muscle. Relative mRNA levels of the indicated genes after 12 weeks of either CD or WD in the (**A**) intestine, (**B**) white adipose tissue (WAT), (**C**) brown adipose tissue (BAT), (**D**) liver, and (**E**) skeletal muscle. The data were normalized to the expression level of the housekeeping β2 microglobulin gene. We used an unpaired Student’s *t*-test (or unpaired *t*-test with Welch’s correction, if variances were unequal) for data analysis, * *p* < 0.05, ** *p* < 0.01, *** *p* < 0.001, **** *p* < 0.0001, ‘ns’ indicates not significant; The data were represented as means ± SEM, *n* = 3–10 samples, derived from *N* = 6 male mice (3 per group, CD and WD). (CD) control diet (black); (WD) Western diet (red).

**Figure 3:**
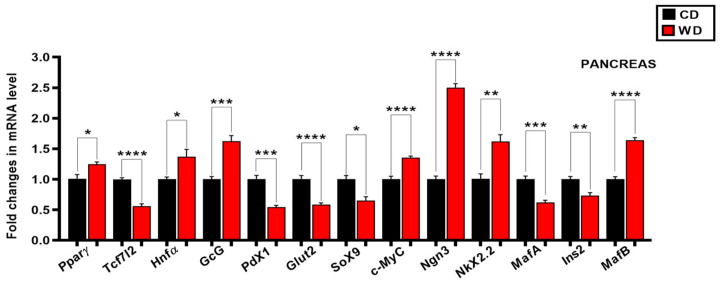
The effect of a 12-week Western diet on the expression of the T2D-associated genes in the pancreatic tissue. Relative mRNA expression levels of the indicated genes in the pancreatic tissue following WD intervention. The data were normalized to the housekeeping β2 microglobulin gene, and expressed as mean ± SEM *n* = 3–9 samples, derived from *N* = 6 male mice (3 per group, CD and WD). * *p* < 0.05, ** *p* < 0.01, *** *p* < 0.001, **** *p* < 0.0001, determined using an unpaired Student’s *t*-test (or unpaired *t*-test with Welch’s correction, if variances were unequal). (CD) control diet (black); (WD) Western diet (red).

To understand the underlying mechanism of WD-induced onset and progression of diabetic phenotype and metabolic dysfunctions, we quantified the expression levels of the candidate genes by quantitative real-time PCR. We analyzed the relative changes in the gene expression in the intestine, adipose tissue, liver, and skeletal muscle (Figure 2), and pancreas (Figure 3) separately. Additional data across all tissues are shown in Supplementary Figure S1.

A striking finding in this study is that the WD induced downregulation of the Wnt signaling gene *Tcf7l2* in the liver (0.72 ± 0.05, *p* = 0.0231 Figure 2D), skeletal muscle (0.64 ± 0.08, *p* = 0.0542, Figure 2E), pancreas (0.56 ± 0.04, *p* < 0.0001, Figure 3), white adipose (0.36 ± 0.02, *p* < 0.0001, Figure 2B) and brown adipose (0.22 ± 0.02, *p* < 0.0001, Figure 2C), whereas in the intestine we observed an upregulation of the same gene (2.57 ± 0.12, *p* < 0.0001, Figure 2A). We also analyzed the expression of *PPARγ*, *Hnf-1α* and *Lrp5,* and we found that WD induced the downregulation of *PPARγ* significantly in the white and brown adipose (0.55 ± 0.05, *p* = 0.0004 and 0.41 ± 0.04, *p* < 0.0001, respectively), (Figure 2B,C), and upregulation in the pancreas (1.25 ± 0.04, *p* = 0.0174, Figure 3), liver (1.46 ± 0.07, *p* = 0.0002, Figure 2D), intestine (1.97 ± 0.10, *p* = 0.0008, Figure 2A) and skeletal muscle (2.59 ± 0.16, *p* < 0.0001, Figure 2E); downregulation of *Hnf-1α* in the liver (0.52 ± 0.04, *p* < 0.0001, Figure 2D), and upregulation in the pancreas and intestine (1.37 ± 0.08, *p* = 0.011 and 2.69 ± 0.13, *p* = 0.0007, respectively), (Figure 2A and Figure 3); downregulation of *Lrp5* in the white and brown adipose (0.39 ± 0.04, *p* < 0.0001 and 0.38 ± 0.03, *p* < 0.0001, respectively), (Figure 2B,C) and upregulation in the intestine (1.72 ± 0.06, *p* < 0.0001, Figure 2A). The expression levels of hepatic and intestinal *Glut2* mRNA were upregulated (1.16 ± 0.04, *p* = 0.0481 and 1.83 ± 0.12, *p* = 0.0021, respectively) (Figure 2A,D); we found the opposite in the pancreatic tissue (Figure 3). In the pancreas we found that the WD induced ectopic expression of the progenitor markers *C-Myc* and *Ngn3* (1.35 ± 0.03, *p* < 0.0001 and 2.50 ± 0.06, *p* < 0.0001, respectively), and decreased the expression of specific adult beta cell genes *Ins2* (0.73 ± 0.05, *p* = 0.0041), *MafA* (0.62 ± 0.03, *p* = 0.0001), *Glut2* (0.58 ± 0.07, *p* < 0.0001) and *Pdx1* (0.54 ± 0.03, *p* = 0.0002); (Figure 3), which can be related to compromised beta cell identity and function. The opposite effects of WD on the upregulations of pancreatic alpha cell identity markers *Nkx2.2* (1.62 ± 0.11, *p* = 0.0037), *GcG* (1.62 ± 0.09, *p* = 0.0008), and *MafB* (1.64 ± 0.04, *p* < 0.0001), were observed (Figure 3). The analysis of the expression of the pluripotent gatekeeper gene *Sox9* in the pancreatic tissue of mice revealed significantly lower mRNA levels in the WD group (0.65 ± 0.06, *p* = 0.0134, Figure 3), suggesting an initiation of endocrine cell differentiation. In striking contrast to the increased expression of *GcG* in the pancreas tissue (Figure 3), the mRNA level of the same gene from the endocrine L cells in the intestinal tissue was not decreased (0.66 ± 0.03, *p* = 0.0128, Figure 2A). Collectively, the RT-qPCR analysis of all the targeted genes (in this study) showed that the gene expression profiles in the targeted tissues (Intestine, WAT, BAT, Liver, Skeletal muscle and Pancreas) of the WD were notably different (Figure 2A–E and Figure 3) as compared to control fed animals (CD) of identical age and initial weights after an identical 12-week feeding period.

## 4. Discussion

In this study, we demonstrated that, compared with the controls, male C57BL/6 mice increase body weight significantly, become insulin resistant, glucose-intolerant, and change expression of diabetes-related gene of interest in the pancreas and other glucose-regulatory tissues after 12 weeks of a WD.

We further demonstrated that WD upregulates both *Glut2* and *Hnf-1α* in the intestine, suggesting that Hnf-1α may act as an additional mechanism promoting increased sugar absorption.

Finally, our results suggest that beta cell dedifferentiation and neogenesis of pancreatic cell lineages occur simultaneously, most likely directed toward non-beta cell types, which may be significant for the development of therapies or dietary interventions aimed at prevention or targeted differentiation toward the desired pancreatic cell lineage.

Moreover, this would be of great significance for further improved understanding and future studies involving a comprehensive investigation of the underlying molecular mechanisms associated with the onset and progression of the disease.

We first targeted intestinal *Glut2* expression, given that previous research has shown that the dysregulation of intestinal *Glut2* mRNA and protein levels brought about by altered insulin action in enterocytes [11,107,108] results in increased glucose absorption from the gut. This leads to acute postprandial hyperglycemia and increases the risk for systemic insulin resistance, hyperinsulinemia, obesity, and diabetes progression [109]. Moreover, Yang et al. showed that the *Glut2* gene is a direct target of HNF-1α in human intestinal cells [110]. However, to date, the relationship between the expressions of these two genes in the small intestine has received insufficient attention. We found that the WD leads to significant upregulation of both *Glut2* and *Hnf-1α* in the intestinal tissue (Figure 2A). Therefore, it seems that increased levels of *HNF-1α* are likely to be an additional underlying mechanism involved in increased sugar absorption from the gut. Further, in individuals with insulin resistance and T2D, the GLP-1 response after a meal is impaired [111]. The exact mechanism underlying this impairment remains poorly understood [112,113,114]. Previous studies have indicated that insulin can regulate the *GcG* gene expression in intestinal endocrine L cells through β-catenin/TCF7L2-dependent transcription [115]. In the present study, we found that the *GcG* mRNA levels in the WD group were decreased, despite increased *Tcf7l2* gene expression (Figure 2A), which suggests an intracellular signaling defect distal to or independent of TCF7L2, or the presence of other more important factors for regulating intestinal *GcG* expression. In this regard, Chiang et al. have suggested that insulin signaling interacts with the Wnt/β-catenin pathway, but does not influence the expression of *GcG* directly [116]. In addition to the above, it is worth considering the possible impact of PPARγ on *GcG* expression. Recently, it has been reported that PPARγ is involved in the modulation of the intestinal *GcG* expression, GLP-1 secretion and SNS (Sympathetic Nervous System) activity related to the stimulation of lipolysis in WAT in mice [117]. More specifically, intestinal PPARγ is believed to affect the expression of GLP-1 negatively. It should be pointed out that *PPARγ* belongs to nutritionally regulated genes, and it is well known that *PPARγ* may be influenced directly by lipids and other dietary components in the small intestine. This may also account for the upregulated *PPARγ* expression in WD mice (Figure 2A). As PPARγ is able to suppress *GcG* expression in mice, we suggest that the increased activation of *PPARγ* gene expression observed in the current study (Figure 2A) possibly leads to dysregulated GLP-1 secretion and, further, exacerbates hyperglycemia.

Taken together, the reduced *GcG* expression in the intestinal tissue observed in our study (Figure 2A) appears to be a consequence of both an insufficient insulin/TCF7L2-mediated transcriptional activation of *GcG* and an increased *PPARγ* expression. Together, our data suggest a vicious circle induced by WD that affects the homeostasis of GLP-1 hormone synthesis.

However, while our findings may suggest a potential functional relevance in the context of understanding the pathophysiology of T2D, functional assays were not performed due to limitations; therefore, these interpretations should be considered preliminary and remain to be validated in future studies.

As to the observed changes in the adipose tissue (Figure 2B,C), the Wnt/β-catenin and the insulin signaling pathway have essential, but opposite, roles in maintaining normal adipose tissue biology. Namely, active Wnt/β-catenin signaling is required to prevent adipocyte differentiation, and thus an increase in the adipose tissue mass, contrary to the role of insulin, which promotes *PPARγ* gene expression, and thus maintains normal adipocyte function, preadipocyte differentiation, and growth [64,65,118]. Chen et al. found that adipocyte insulin resistance in humans is associated with a reduction in *Tcf7l2* mRNA expression [66]. In line with this, a conditional deletion of *Tcf7l2* in adipocytes in mice exposed to HFD leads to insulin resistance and adipocyte hypertrophy, enhanced weight gain, and an impaired glucose and lipid metabolism [119]. Consistent with the above notions, decreased *Tcf7l2* gene expression in the current study suggests a hypertrophic and insulin-resistant diabetic adipose tissue. Interestingly, during preadipocyte differentiation, a cross-talk between insulin and Wnt signaling via the Wnt/β-catenin LRP5 co-receptor has been demonstrated. From this, it seems that *LRP5* has an essential and dual role in both signaling pathways. Thus, the decrease in *LRP5* expression found in this study suggests a negative effect on new adipocyte formation in WD mice. Moreover, the inability of WAT to expand through the differentiation of preadipocytes into mature adipocytes due to overnutrition may lead to adipocyte hypertrophy and accumulation of excess lipids in non-adipose tissues [120]. PPARγ represents the adipocyte master transcriptional regulator [36]. According to previous studies, the impaired differentiation of adipose tissue and a compensatory adipocyte hypertrophy, caused by lower expression of *PPARγ*, as well as a change in the adiponectin production and secretion, can increase TNF-α activity and contribute further to chronic adipose tissue inflammation and insulin resistance [121,122,123,124]. The downregulation of *PPARγ* gene expression in the current study thus suggests an impairment of adipogenesis, inflammation, and limitation of WAT to store triglycerides properly. Based on the additional functional roles of PPARγ in the BAT [125], the observed pathological changes in *PPARγ* gene expression in BAT in our study further indicate a disturbance in the stimulation of energy expenditure and impaired BAT functionality. To the best of our knowledge, this is the first report that WD affects the expression of targeted genes negatively in both types of adipose tissue (Figure 2B,C). Taken together, our data indicate that adipose tissue function deteriorates with a 12-week WD. However, further studies are required to clarify the underlying mechanisms, which may contribute importantly to the development of the observed diabetic phenotype.

In the liver, both Wnt/β-catenin and insulin signaling regulate hepatic gluconeogenesis negatively [104]. In mouse hepatocytes, insulin induces β-catenin Ser675 phosphorylation, consequently amplifying TCF7L2 activation and repressing gluconeogenic gene expression. Ip et al. also demonstrated that insulin treatment tends to increase hepatic *Tcf7l2* mRNA levels. However, the expression profile of the *Tcf7L2* gene is unknown in the liver of WD mice. Concurrently, in the present study, we detected decreased mRNA levels (Figure 2D) of this key T2D gene. Our finding is also consistent with a previous report that TCF7L2 expression was downregulated in the livers of obese T2D mice on an HFD [126], suggesting that chronic insulin resistance may lead to downregulation of *Tcf7l2* gene expression. Consequently, these observations, collectively, suggest that IR provoked by WD increases hepatic gluconeogenesis, at least partly through the downregulation of *Tcf7l2*.

Furthermore, the upregulation of *PPARγ* induced by HFD has been described to lead to excessive hepatic lipid accumulation, and to be one of the underlying mechanisms of liver dysfunction, and progression to hepatic steatosis and T2D [127]. In contrast, HNF-1α has been reported to prevent excessive deposition of hepatocyte fat [128]. Our data demonstrate decreased *HNF-1α* mRNA levels (Figure 2D), which suggests the development of hepatic inflammation and liver fibrosis [129]. Our data are supported by the previous finding that hepatocyte-specific HNF-1α deficiency in HFD mice results in elevated levels of *PPARγ*, *TNFα*, *IL-6* mRNA, and hepatic lipid accumulation [130]. Although we did not measure the expression levels of inflammatory markers, we also found significantly increased *PPARγ* expression, which is a strong indicator of hepatic IR and compromised liver function (Figure 2D).

In this study, we also noted that the levels of *Glut2* mRNA in the liver were up-regulated (Figure 2D), indicating a facilitated hepatic glucose uptake and possibly increased liver lipogenesis. Previously, it has been well established that the accumulation of lipids initiates hepatic steatosis and liver dysfunction. It should also be noted that the regulation of hepatic *Glut2* gene expression in rodents is under PPARγ [131], glucose, and insulin control [132]. Interestingly, a recent study provided direct evidence that HNF-1α is a transcriptional repressor of *PPARγ* in the liver steatosis-associated cancer models, highlighting a mechanism that seems to be conserved evolutionarily in both mice and humans [133]. The results from our present study, with a combination of markedly increased *PPARγ* and decreased *HNF-1α*, indicate strongly that WD feeding results in a disturbance of this evolutionarily conserved and fine-tuned mechanism regulating a balanced hepatic lipid flux. Moreover, HNF-1α is involved directly in the regulation of hepatic cholesterol metabolism and homeostasis [24]. Hence, our data are consistent with previous reports on changes in the expression profiles of key candidate genes in the liver during the development of T2D, which may underlie a marked dysregulation in hepatic glucose and lipid metabolism.

While our findings align with previous studies using high-fat diet (HFD) mouse models that poorly mimic the complexity of human T2D, our study utilized a more physiologically relevant model for investigating disease mechanisms, i.e., a Western diet, which better replicates human T2D pathophysiology in mice. Results revealed altered hepatic *HNF-1α* expression, thereby identifying *HNF-1α* as a promising molecular target for the development of future therapies aimed at preventing liver dysfunction and, consequently, may open new avenues for ameliorating diabetes and related severe metabolic conditions.

The Wnt/β-catenin signaling plays multiple essential roles in the maintenance of mammalian skeletal muscle homeostasis [134,135,136] and the balance between myogenic and adipogenic potential in adult myoblasts [137]. Thus, disturbances in this evolutionary conserved signaling pathway in the skeletal muscle may have an impact on the whole-body energy balance. Moreover, it is important to highlight that the Wnt/β-catenin downstream effector TCF7L2 is associated with IR. More specifically, it has been identified as a transcriptional regulator of the insulin receptor, and this mechanism is evolutionarily conserved between species [138]. Recently, Kupczewska et al. have shown that the skeletal muscle Wnt signaling-associated genes, including *Tcf7l2* gene expression, were up-regulated in young and healthy male human subjects with low IS [139], suggesting that enhanced Wnt signaling pathway activation may be a compensatory mechanism for enhancing glucose uptake into muscles during early changes in IS. Interestingly, they have also demonstrated, both in vitro and in vivo, that hyperinsulinemia regulated the expression profile of the Wnt/β-catenin related genes positively, including the expression of *Tcf7l2* in skeletal muscles. However, FFAs abolished this effect. In contrast to the study by Kupczewska et al., which was performed on bioptic muscle samples from young healthy men, we examined the mRNA levels of *Tcf7l2* in the skeletal muscle of insulin-resistant diabetic mice, and demonstrated an opposite pattern of *Tcf7l2* gene expression, as shown in Figure 2E. Clearly, further studies are needed to track changes in *Tcf7l2* expression longitudinally during the development of diabetes, but it seems that dysregulation of *Tcf7l2* expression may be an early marker for defective insulin signaling, and even contribute to the development of the skeletal muscle IR seen in T2D. Moreover, Park et al. reported that the *PPARγ* gene expression is elevated in skeletal muscle in T2D [140], and data from our current study support this finding (Figure 2E). The up-regulated *PPARγ* expression could have a protective role in protecting the body from skeletal muscle IR. Namely, Kruszynska et al. have found that, while *PPARγ* expression does not necessarily differ between the diabetic and control subjects, and does not increase upon short-term hyperinsulinemia, it seems to correlate with the percentage of body fat in normoglycemic obese subjects [141]. In addition, Amin et al. demonstrated that selective activation of PPARγ in skeletal muscles induces endogenous production of adiponectin and protects transgenic mice on the C57BL/6J background from HFD-induced IR [142]. Taken together, these previous investigations support the notion that the activation of *PPARγ* in pathophysiological states could help maintain skeletal muscle IS. However, while PPARγ probably promotes fat oxidation in skeletal muscles [143,144,145], Muoio et al. have raised the interesting possibility that this increased oxidation may not necessarily be coupled to the downstream tricarboxylic acid (TCA) cycle and electron transport chain, facilitating accumulation of disruptive incompletely oxidized metabolites [146]. Based on the above and our findings of decreased *Tcf7l2* and increased *PPARγ* expression, it seems that, at least in our animal model and at the studied time point during the development of diabetes, skeletal muscles are still at least compensating partly for the developing IR, and that the compensatory pathway via *Tcf7l2* may be disrupted sooner than the one via *PPARγ*. However, at present, this is a hypothesis that needs to be clarified in future studies by analyzing more time points during the development of muscle IR, and also looking at the *PPARγ* coactivator-1 (PGC1), which also seems to be able to increase the downstream degradation of fatty acids.

Here, given that we demonstrated for the first time that a Western diet disturbs *Tcf7l2* gene expression in mouse skeletal muscle, further investigation into the underlying molecular mechanisms is warranted, as this could pave the way for targeted, muscle-specific therapies in T2D.

Additionally, taking into account *a novel* integrative approach developed for potential biomarker discovery and disease prediction, we conducted a bioinformatics analysis based on an interesting recent study [147], comparing the target genes from our study with human gene homologs, focusing particularly on differentially expressed genes [148]. However, this analysis did not reveal a significant overlap (likely due to various factors, such as small cohort sizes, the differences between humans and mice), highlighting the complexity of tissue-specific responses in diabetes and underscoring the need for broader, integrative approaches in future research.

To gain insight into the impact of WD on the overall pancreas function, we measured the mRNA levels of specific pancreatic adult mouse alpha and beta cells’ gene markers. MAFA is essential in maintaining adult beta cell identity and function in mice. Namely, it controls *Ins1*, *Ins2*, *Pdx1,* and *Glut2* gene expression, and, thus, insulin biosynthesis and glucose-stimulated insulin secretion (GSIS) in fully functioning mature beta cells [149]. Interestingly, during embryogenesis, MAFB has an essential role in the stimulation of *Ins*, *Pdx1*, and *Glut2* gene transcription (before the onset of *MafA* expression at E13.5), but postnatally, it becomes restricted to islet alpha cells [150]. Namely, contrary to the prenatal beta cells, MAFB is a key regulator of glucagon production and secretion in mouse pre- and postnatal alpha cells [151,152]. However, it is important to note that data from the Xiafukaiti et al. study [153] revealed potential evidence for a unique functional role of MAFB in maintaining mature beta cell features under some specific conditions.

We observed significant alterations in the expression of all the examined genes associated with normal adult beta cell function, i.e., *Ins2*, *Pdx1*, *MafA,* and *Glut2* (Figure 3). On the other hand, the mRNA levels of the specific adult alpha cell markers *GcG* and *MafB* were regulated in the opposite direction (Figure 3). In total, these findings provide strong evidence that WD feeding induced significant alterations in the insulin and counter-regulatory hormone glucagon encoding *GcG* gene expression, and the alarming shift in the expression profile of other key functional mature alpha and beta cell genes. Thus, our in vivo data indicate a disturbance of balanced bi-hormonal islet regulation (a well-recognized phenomenon in the pathogenesis of T2D), increased alpha cell activity or mass, functional beta cell failure, and, as a consequence, glucose homeostasis disruption and metabolic deregulation in WD mice.

Moreover, the observed downregulation of specific adult beta cell markers, including the *Ins2* gene expression, is typical for early phases of T2D onset and progression, and in response to insulin resistance (IR), may be considered, together with beta cell dedifferentiation, as a compensatory functional adaptation in the context of preserving the functionality of pancreatic beta cells from exhaustion and eventual damage under chronic hyperinsulinemic conditions [1,154].

Although direct measurement of GLP-1 secretion from intestinal endocrine L cells presents significant methodological challenges [155], and insulin levels were not measured in this study, we have previously demonstrated that WD-fed male C57BL/6J mice exhibit hyperinsulinemia [156], providing indirect evidence of the metabolic stress imposed by this dietary type. This further supports our decision to focus on transcriptional changes across glucose-regulatory tissues in mice.

The Wnt/β-catenin signaling and its downstream component TCF7L2 are involved potently in pancreas development, islet function, insulin production, and secretion [157]. Previous observations suggest that the normal physiological activity of the Wnt/β-catenin signaling is essential for preserving and maintaining adult pancreatic cells in non-pathophysiological conditions. This is supported by the finding that *Tcf7l2* silencing resulted in a marked reduction in the key genes involved in the maintenance of proper mature pancreatic beta cell function, proinsulin synthesis, and processing [158]. Our data demonstrate that *Tcf7l2* gene expression is downregulated in WD-fed mice (Figure 3). This finding would suggest functional defects of beta cells, related specifically to decreased insulin secretion, and, consequently, insufficient compensation in maintaining proper beta cell function, but future studies of beta cell function in WD mice, assessed by electrophysiology, calcium imaging, or secretion measurements, will be needed to clarify this further.

In this study, we also noted increased *PPARγ* gene expression in pancreatic tissue (Figure 3), which has a detrimental effect on pancreatic beta cells. More specifically, it has been shown previously that increased expression of *PPARγ* in the islet beta cells can induce negative effects on beta cell function, including increased lipid accumulation, and, thus, cause a lipotoxic beta cell phenotype, cellular stress, followed by increased beta cell apoptosis and decreased insulin production and GSIS in the HFD male mice [159]. Thus, our results suggest that beta cell dysfunction in WD diabetic mice could also be induced and associated with hyperlipidemia, excess fatty acid accumulation, beta cell stress, and consequent apoptosis, which exacerbates T2D.

The transcription factor HNF-1α plays an important role in the function of adult pancreatic alpha and beta cells. Namely, it is essential for the regulation of *Glut2* expression in the pancreatic beta cells, and, very recently, it has been shown that HNF-1α controls glucagon secretion in pancreatic alpha cells in mice [160]. Here, it should be emphasized that heterozygous mutations in human *HNF-1α* (MODY3) are associated strongly with monogenic forms of diabetes in young people (maturity-onset diabetes of the young), which is manifested presumably by the loss of glucose sensing, insulin secretion disorder, and, thus, defects in beta cell functions [161]. Given its substantial role in maintaining normal physiological alpha and beta cell functions, in the present study, we showed upregulation of *HNF-1α* mRNA expression in the pancreatic tissue of the WD mice (Figure 3). The results from this study, together with data obtained from others, suggest that pancreatic *HNF-1α* overexpression may disturb both the glucagon secretion in the alpha cells and *Glut2* expression in the pancreatic beta cells, and, thus, compromise the normal function of both islet cell types.

The mature state of the beta cell can be perturbed due to exposure to distinct stressors, including de novo activation of “disallowed” endocrine progenitor marker expression, and this results in the loss of their cellular identity and function [162]. However, the mechanisms linking the overall impact of WD on beta cell lipotoxicity, abnormal beta-cell function, and dedifferentiation remain largely unknown. In this study, we found that genes that are normally not expressed or expressed at very low levels in the adult pancreas, i.e., *Ngn3* and *c-Myc,* are expressed more highly in WD-fed mice (Figure 3). In line with this, increased *c-Myc* expression in our current study (Figure 3) suggests both beta cell apoptosis and dedifferentiation. The activation of *c-Myc* in adult beta cells results in impaired insulin secretion and a reduction in the functional beta cell mass [163]. Furthermore, it has been demonstrated that aberrant expression of *c-Myc* alone in adult beta cells compromises the expression of important function-maintaining genes, thus leading to the loss of beta cell differentiation, and diverting beta cells toward a less-mature phenotype [164]. However, here, it should be pointed out that increased *MafB* expression in the current study may also indicate beta-cell dedifferentiation. Ectopically increased expression of the immature beta cell/adult alpha cell marker *MafB* (which is non-specific for mature beta cells) and the “disallowed” progenitor genes *Ngn3* and *c-Myc* in this study, suggests an in vivo lineage reprogramming, which might lead to a change in beta cell identity, the decline in the number of mature functional insulin-producing beta cells, and beta cell apoptosis. However, further studies addressing these possibilities directly are needed to clarify their role in T2D pathogenesis under WD.

Recently, the pancreatic duct has been identified as a potential source of new beta cells [165,166,167]. Does beta cell neogenesis occur under conditions of overnutrition in mice as a possible mechanism to compensate for the elevated blood glucose levels? To at least address this question partly, we measured the levels of *Sox9* mRNA expression (a marker of the progenitor pool in the pancreatic ducts), and we showed here that they are reduced in the pancreatic tissue obtained from WD-fed mice (Figure 3). Interestingly, during pancreatic development, TCF7L2 regulates NGN3-mediated *Nkx2.2* expression negatively, but overexpression of *Ngn3* reduces both the *Tcf7l2* mRNA and protein levels, and induces activation of the *Nkx2.2* gene expression in a mouse duct cell line [168]. In line with this, the repressor activities of NKX2.2 are sufficient for the differentiation of the alpha cell type in mice. Moreover, our in vivo data demonstrate that WD increased pancreatic *Nkx2.2* gene expression significantly (Figure 3). Our data, and previously described data together, suggest that ductal cell reprogramming may be an additional significant pathophysiological contributing mechanism for insufficient insulin production (the deficit in functional beta cell mass) and perturbed islet function in WD mice. Given the experimental limitations in this study, these results are difficult to interpret. Nevertheless, if we take into consideration the fact that dedifferentiation, dysfunction, and beta cell loss per se are implicated in the pathogenesis of T2D, we demonstrate here the possible existence of all three processes, which is based on the pattern of specific gene expression. Taken together, these results extend our understanding of how lifestyle changes impact and induce alpha cell function alteration and beta cell failure, stress, destruction, and loss.

## 5. Conclusions

Our paper provides the first comprehensive landscape of systemic, multi-tissue early transcriptional changes in T2D-related genes under Western diet (WD) conditions, in male C57BL/6 mice after 12 weeks of WD, offering novel insights into mechanisms that may underlie WD-induced T2D onset and the early events of disease development and progression. Previously, gene expression in only two or three tissues or cell types in high-fat diet (HFD) mouse models was reported. In addition, we believe that the WD model more accurately mimics the human dietary pattern than previously studied high-fat diet (HFD) models alone, which makes our findings in WD mice more relevant to human type 2 diabetes (T2D). We showed that WD induces obesity, impairs glucose homeostasis, and triggers tissue-specific dysregulation of key metabolic genes. Western diet (WD) induces coordinated, tissue-specific transcriptional changes across the intestine, adipose tissue, liver, skeletal muscle, and pancreas, disrupting key regulators of glucose handling, insulin sensitivity, and islet identity. We reveal novel mechanisms, including *Hnf–1α*-mediated *enhancement* of intestinal sugar absorption, the first evidence of differential regulation of *Tcf7l2,* including its downregulation in skeletal muscle, and early islet reprogramming in WD models.

Despite limitations, including the lack of functional experiments, the small number of animals, and the use of RNA from the whole pancreas, these findings provide important and potentially significant insights into the mechanisms underlying early disease onset and progression. Together with previous studies, our results advance the understanding of molecular mechanisms involved in T2D pathogenesis and support public health efforts promoting a healthy diet to prevent T2D and associated complications.

## Figures and Tables

**Table 1:** Composition of the CD (control diet) and WD (Western diet) used in the study.

Nutritional Ingredients	CD	WD
Arachidonic acid, %	0.2	0.0
Carbohydrates, %	60.1	50.0
Cholesterol, %	0.018	0.21
Essential amino acids	5.5	7.3
Fat, %	4.5	21.0
Fiber, %	4.9	0.2
Linoleic acid, %	0.19	1.48
Minerals, %	2.65	0.04
Monounsaturated fatty acids, %	23.0	12.6
Nonessential amino acids	8.0	12.8
Polyunsaturated fatty acids, %	53.8	2.86
Proteins, %	14.5	20.0
Saturated fatty acids, %	22.0	25.8
Vitamins, %	<5	0.01
Water, %	<11	<9
Energy profile		
Carbohydrates, % of kcal	71.7	43.0
Energy, kcal/g	3.0	4.7
Fat, % of kcal	10.5	40.0
Proteins, % of kcal	40.0	17.0

## Data Availability

The original contributions presented in this study are included in the article/Supplementary Material. Further inquiries can be directed to the corresponding authors.

## Supplementary Materials

The following supporting information can be downloaded at https://www.mdpi.com/article/10.3390/cimb47121053/s1.

## Abbreviations

The following abbreviations are used in this manuscript:

*β2M*	Beta 2 microglobulin
ANOVA	Analysis of variance
AUC	Area under the curve
BAT	Brown adipose tissue
CD	Control diet
cDNA	Complementary Deoxyribonucleic acid
*c-Myc*	Myc myelocytomatosis oncogene
CO_2_	Carbon dioxide
Ct	Cycle threshold value
FFAs	Free fatty acids
*GcG*	Glucagon
GLP-1	Glucagon-like peptide-1
*Glut2*	Glucose transporter 2
GSIS	Glucose stimulated insulin secretion
GWAS	Genome-wide association studies
HFD	High-fat diet
*Hnf-1α*	Hepatocyte nuclear factor-1 alpha
*IL-6*	Interleukin-6
*Ins2*	Insulin 2
ipITT	Intraperitoneal insulin tolerance test
ipGTT	Intraperitoneal glucose tolerance test
IR	Insulin resistance
IS	Insulin sensitivity
*Lrp5*	Low-density lipoprotein receptor-related protein 5
*MafA*	MAF bZIP transcription factor A
*MafB*	MAF bZIP transcription factor B
MODY3	Maturity-onset diabetes of the young 3
mRNA	Messenger Ribonucleic acid
*Ngn3*	Neurogenin 3
*Nkx2.2*	NK2 Homeobox 2
NOD	Non obese diabetic
PBS	Phosphate-buffered saline
PCR	Polymerase chain reaction
*Pdx1*	Pancreatic and duodenal homeobox 1
PGC-1α	PPARγ coactivator-1 alpha
*PPARγ*	Peroxisome proliferator activated receptor gamma
RT-qPCR	Quantitative real-time polymerase chain reaction
SEM	The standard error of the mean
*Slc2a2*	Solute carrier family 2 member 2 (*Glut2*)
SNS	Sympathetic nervous system
*Sox9*	SRY (Sex determining region Y)-box 9
T2D	Type 2 diabetes
TCA	Tricarboxylic acid cycle
*Tcf7L2*	T cell-specific transcription factor 7-like 2
*TNFα*	Tumor necrosis factor alpha
WAT	White adipose tissue
WD	Western diet
Wnt	Wingless-type MMTV integration site family
