# Supplementary material for: Western Diet Induces Changes in Gene Expression in Multiple Tissues During Early Insulin Resistance and Glucose Intolerance in Male C57BL/6 Mice
Source: Curr Issues Mol Biol. 2025 Dec 16;47(12):1053. doi: 10.3390/cimb47121053 (PMC12731596; doi:10.3390/cimb47121053)
Supplement: Supplementary file 1 [file cimb-47-01053-s001.zip › cimb-4012945-supplementary.pdf]

Supplementary Material

Western diet induces changes in gene expression in multiple tissues during early insulin resistance and glucose intolerance in male C57BL/6 mice

Supplementary Table S1. List of genes and primers sequences (Species-*Mus musculus*) used in the study for relative gene expression level analysis by real-time quantitative PCR (RT-qPCR)

Gene	Name	Forward primer sequence	Reverse primer sequence	Ref
β2M	Beta 2 microglobulin (B2m)	CATGGCTCGCTCGGTGAC	CAGTTCAGTATGTTCGGCTTCC	[92]
C-MyC	Myc myelocytomatosis oncogene	CAAATCCTGTACCTCGTCCG	GTTGTGCTGGTGAGTGGAGA	[62]
GcG	Glucagon	ACAGAGGAGAACCCCAGATC	CATCATGACGTTTGGCAATG	[93]
Glut2	Glucose transporter 2	TTCCAGTTCGGCTATGACATCG	CTGGTGTGACTGTAAGTGGGG	[94]
Hnfα	Hepatocyte nuclear factor 1α	AGAGCCCCTTCATGGCAACC	TGAAGACCTGCTTGGTGGGTG	[95]
Ins2	Insulin 2	CCTGCTGGCCCTGCTCTTC	GCTTCTGCTGGGCCACCTC	[93]
Lrp5	Low-density lipoprotein receptor-related protein 5	AAGCAACAGTGTGACTCCTTC	GGGAGAGGATGATACCAATGAC	[96]
MafA	MAF bZIP transcription factor A	TTCAGCAAGGAGGAGGTCAT	CCGCCAACTTCTCGTATTTC	[97]
MafB	MAF bZIP transcription factor B	TGAGCATGGGGCAAGAGCTG	CCATCCAGTACAGGTCCTCG	[98, 99]
Ngn3	Neurogenin 3	CTCCGACCATCCATCACTTT	GCAGAAGAAGGCAGATCACC	[100]
Nkx2.2	NK2 Homeobox 2	GGTGGAGCGATTGGATAAGA	TGCCATCAACCTTTTCATCA	[101, 102]
Pdx1	Pancreatic and duodenal homeobox 1	CATCTCCCCATACGAAGTGC	GGGGCCGGGAGATGTATTTG	[97]
PPARγ	Peroxisome proliferator activated receptor gamma	TGTCGGTTTCAGAAGTGCCTTG	TTCAGCTGGTCGATATCACTGGAG	[92]
Sox9	SRY (sex determining region Y)-box 9	ACAGATCTCCTACAGCCCCTTCAA	GCCGGAGTTCTGATGGTCAGCGTA	[103]
Tcf7L2	Transcription factor 7 like 2	GCATCCCTCACCCGGCCATC	GCCACCTGCGCCCGAGAATC	[104]

The primer sequences used for RT-qPCR were obtained from professional scientific publications or generated using Primer-BLAST [100], as appropriate.

Supplementary Table S2. Composition of nutritional ingredients and energetic profiles of the CD (control diet) and a WD (Western diet) used in the study

Composition of diets	CD	WD
**Nutritional ingredients**		
Arachidonic acid, %	0.2	0.0
Carbohydrates, %	60.1	50.0
Cholesterol, %	0.018	0.21
Essential amino acids	5.5	7.3
Fat, %	4.5	21.0
Fiber, %	4.9	0.2
Linoleic acid, %	0.19	1.48
Minerals, %	2.65	0.04
Monounsaturated fatty acids, %	23.0	12.6
Nonessential amino acids	8.0	12.8
Polyunsaturated fatty acids, %	53.8	2.86
Proteins, %	14.5	20.0
Saturated fatty acids, %	22.0	25.8
Vitamins, %	<5	0.01
Water, %	<11	<9
**Energy profile**		
Carbohydrates, % of kcal	71.7	43.0
Energy, kcal/g	3.0	4.7
Fat, % of kcal	10.5	40.0
Proteins, % of kcal	40.0	17.0

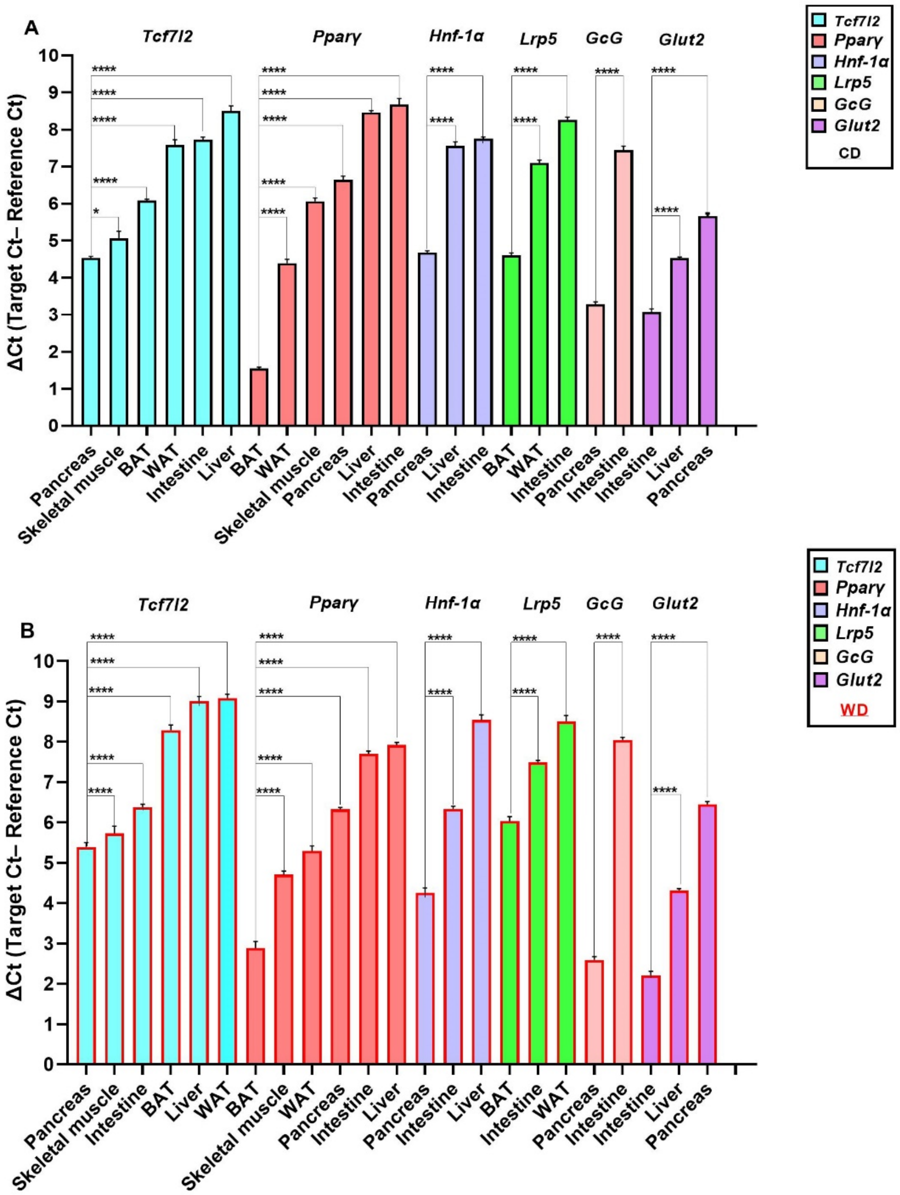

Supplementary Figure S1. Expression levels of targeted diabetes related genes (*Tcf7l2*, *PPARγ*, *HNF-1α*, *Lrp5*, *GcG* and *Glut2*) during insulin resistance in different tissues based on ΔCt values in (A) CD (black) and (B) WD (red) fed mice. Bar graph showing raw ΔCt values of targeted genes, measured by RT-qPCR in: intestine, white adipose tissue (WAT), brown adipose tissue (BAT), liver, skeletal muscle and pancreas after 12 weeks of either CD or WD. ΔCt values were calculated as the difference between the Ct value of the target gene and the Ct value of the reference, housekeeping the β2 microglobulin, gene. We used unpaired Student's t-test for data analysis, *$p < 0.05$, ****$p < 0.0001$; Data were represented as means ± SEM, n = 3 male mice for each group. **(A)** (CD) control diet (black); **(B)** (WD) Western diet (red). Note, lower ΔCt values indicate higher gene expression relative to internal control.

In CD fed mice, *Tcf7l2* had a significantly lower expression in skeletal muscle (1.12 ± 0.17-fold decrease, $p < 0.05$), BAT (1.34 ± 0.05-fold decrease, $p < 0.0001$), WAT (1.67± 0.12-fold decrease, $p < 0.0001$), intestine (1.71

± 0.07-fold decrease, p < 0.0001) and liver (1.88 ± 0.12-fold decrease, p < 0.0001), compared to pancreas where TCF7L2 promotes normal pancreatic beta cell functions (Figure S1A); downregulation of *PPARγ* in WAT (2.85 ± 0.10-fold decrease, p < 0.0001), skeletal muscle (3.93± 0.09-fold decrease, p < 0.0001), pancreas (4.31 ± 0.11-fold decrease, p < 0.0001), liver (5.49± 0.07-fold decrease, p < 0.0001) and intestine (5.63 ± 0.15-fold decrease, p < 0.0001) compared to BAT (Figure S1A) where PPARγ orchestrates the expression of genes required for brown fat cell identity and physiological functions such as, lipid metabolism and thermogenesis; *Hnf-1α* shows lower expression levels in liver (1.62 ± 0.12-fold decrease, p < 0.0001) and intestine (1.66 ± 0.07-fold decrease, p < 0.0001) compared to pancreas where it plays a crucial role (Figure S1A); relative to BAT, *Lrp5* shows reduced expression in WAT (1.54 ± 0.10-fold decrease, p < 0.0001) and intestine (1.79 ± 0.09-fold decrease, p < 0.0001) (Figure S1A) than in BAT (Figure S1A); the expression level of *GcG* is evidently lower in intestinal tissue (2.27 ± 0.11-fold decrease, p < 0.0001) than in pancreatic tissue (Figure S1A) and the expression level of *Glut2* is evidently higher in intestinal tissue than in hepatic (1.47 ± 0.09-fold decrease, p < 0.0001) and pancreatic tissue (2.29 ± 0.11-fold decrease, p < 0.0001) of mice fed a control diet (CD) (Figure S1A).

In diabetic samples isolated from WD mice, *Tcf7l2* expression was a significantly lower in skeletal muscle (1.06 ± 0.19-fold decrease, p < 0.0001), intestine (1.18 ± 0.14-fold decrease, p < 0.0001), BAT (1.54 ± 0.17-fold decrease, p < 0.0001), liver (1.67 ± 0.16-fold decrease, p < 0.0001) and WAT (1.68 ± 0.15-fold decrease, p < 0.0001) where it exhibits the lowest activity compared to pancreatic tissue (Figure S1A). Thus, analysis of ΔCt values revealed a tissue-specific pattern of *Tcf7l2* expression further suggesting that *Tcf7l2* may play a more prominent regulatory role in pancreatic tissue, where influencing insulin gene expression and normal pancreatic beta cell function. On the other hand, given its known association with T2D the descending expression observed in peripheral metabolic tissues i.e. muscle, intestine, brown adipose, liver and WAT further supports its involvement in peripheral insulin sensitivity and glucose homeostasis during disease progression. Thus, these findings align with its role that plays in T2D pathophysiology.

The relatively low expression of *PPARγ* in skeletal muscle (1.63 ± 0.26-fold decrease), WAT (1.83 ± 0.22-fold decrease, p < 0.0001), pancreas (2.19 ± 0.22-fold decrease, p < 0.0001), intestine (2.67 ± 0.25-fold decrease, p < 0.0001) and liver (2.75 ± 0.17-fold decrease, p < 0.0001) compared to BAT (Figure S1B) where *PPARγ* plays a key role by regulating brown adipocyte differentiation and physiological functions such as lipid metabolism and thermogenesis. Thus, suggests that *PPARγ* maintains a thermogenic and lipid metabolic processes in BAT even under disease conditions. On the other hand, the expression of *PPARγ* in skeletal muscle and WAT may reflect a compensatory mechanism in an attempt to preserve insulin sensitivity and lipid storage in peripheral tissues. Considering *PPARγ* functional role and that *PPARγ* was more highly expressed in BAT than in other tissues (Figure S1B), these differences further indicate that *PPARγ* may play distinct roles across tissues in response to disease progression. Thus, possibly further contributing to disrupted glucose and lipid homeostasis seen in WD mice.

In contrast to the highest expression of *Hnf-1α* in the pancreas the expression of this gene is lower in the intestine (1.49 ± 0.17-fold decrease, p < 0.0001) and liver (2.01 ± 0.18-fold decrease, p < 0.0001) (Figure S1B). When compared to CD (Figure S1A), the pancreas still remains the primary site with the highest expression levels of this gene in absolute terms (Figure S1A-B), which further implies that the next most prominent site of increased expression (i.e; dysregulation) under a diabetic condition, as a result of consuming a Western-style diet, occurs in the intestine. This may indicate a new or enhanced role for *Hnf-1α* in the intestinal tissue during disease onset and progression in individuals with diabetes.

Conversely, expression of the Wnt signaling gene *Lrp5* is lower in the intestine (1.32 ± 0.13-fold decrease, p < 0.0001) (Figure S1B) and WAT (1.41 ± 0.18-fold decrease, p < 0.0001) (Figure S1B) relative to its expression observed in BAT, suggesting that WD differentially affecting the *Lrp5* gene expression in peripheral tissues. Thus, under diabetic conditions, a relative higher level of its expression (Figure S1B), may represent a compensatory response aimed at maintaining lipid storage in adipose tissue. According to the well-known role it plays in the regulation of adipocyte differentiation and energy metabolism in both types of adipose tissue, disturbed *Lrp5* likely contributes to impaired lipid storage and insulin resistance, which are hallmarks of T2D progression. On the other hand, as a consequence, maintaining a higher level of expression in the intestine (Figure S1B) may potentially increase the activity of the Wnt/β-catenin signaling, which could lead to enhanced proliferation of intestinal epithelial cells or affect the secretion of incretin hormones, factors especially important in the context of T2D.

Furthermore, altered *GcG* expression in pancreatic as well as the associated reduction in expression in the intestinal tissues (3.11 ± 0.18-fold decrease, p < 0.0001) of insulin-resistant and diabetic mice fed a Western-style diet (WD) (Figure S1B) may negatively impact systemic glucose homeostasis. Given that *GcG* regulates glucagon production in pancreatic alpha cells and counter-regulatory incretin hormones such as GLP-1 in intestinal L-cells, these findings, at the same time, highlight the importance of monitoring its expression across those metabolic tissues during disease onset and progression to support the development of targeted therapies with the aim of treating T2D.

Finally and in agreement with its well established activity i.e. expression in individuals with T2D, in a WD fed mice we demonstrated that the *Glut2* gene expression was highest in peripheral glucose homeostatic tissues i.e. intestine (2.21-fold) and the liver (1.95 ± 0.09 -fold decrease, p < 0.0001, Figure S1B), in contrast to the pancreas, in which its expression was significantly lower relative to its expression level observed in the intestine (2.92 ± 0.12-fold decrease, p < 0.0001) (Figure S1B). Taking into account that *Glut2* (encode for GLUT2 facilitative glucose transporter) expression exhibited a clear tissue-specific pattern, the observed reduction in the *Glut2* gene expression in pancreatic tissue, compared to relative higher levels observed in the liver and especially intestinal tissue, suggest increased intestinal glucose absorption and hepatic output in mice fed a WD, both factors strongly contribute to elevated blood glucose levels seen in diabetic individuals. While this aberrant pattern is common in diabetic individuals, it should be emphasized that the reduced expression of the *Glut2* gene in pancreatic tissue is a clear sign of beta cell dysfunction, a hallmark of T2D.

References

62. Columbus, J., et al., Insulin treatment and high-fat diet feeding reduces the expression of three Tcf genes in rodent pancreas. J Endocrinol, 2010. 207(1): p. 77-86, doi:10.1677/JOE-10-0044

92. Gong, H., et al., *Evaluation of candidate reference genes for RT-qPCR studies in three metabolism related tissues of mice after caloric restriction.* Sci Rep, 2016. **6**: p. 38513, doi:10.1038/srep38513.

93. Oshima, Y., et al., *Isolation of mouse pancreatic ductal progenitor cells expressing CD133 and c-Met by flow cytometric cell sorting.* Gastroenterology, 2007. **132**(2): p. 720-32, doi:10.1053/j.gastro.2006.11.027.

94.. Taylor, B.L., F.F. Liu, and M. Sander, *Nkx6.1 is essential for maintaining the functional state of pancreatic beta cells.* Cell Rep, 2013. **4**(6): p. 1262-75, doi:10.1016/j.celrep.2013.08.010.

95. Martovetsky, G., J.B. Tee, and S.K. Nigam, *Hepatocyte nuclear factors 4alpha and 1alpha regulate kidney developmental expression of drug-metabolizing enzymes and drug transporters.* Mol Pharmacol, 2013. **84**(6): p. 808-23, doi:10.1124/mol.113.088229.
96. Badders, N.M., et al., *The Wnt receptor, Lrp5, is expressed by mouse mammary stem cells and is required to maintain the basal lineage.* PLoS One, 2009. **4**(8): p. e6594, doi:10.1371/journal.pone.0006594.
97. Matsuoka, T.A., et al., *Regulation of MafA expression in pancreatic beta-cells in db/db mice with diabetes.* Diabetes, 2010. **59**(7): p. 1709-20, doi:10.2337/db08-0693.
98. Takeuchi, T., et al., *Neither MafA/L-Maf nor MafB is essential for lens development in mice.* Genes Cells, 2009. **14**(8): p. 941-7, doi:10.1111/j.1365-2443.2009.01321.x.
99. Moriguchi, T., et al., *MafB is essential for renal development and F4/80 expression in macrophages.* Mol Cell Biol, 2006. **26**(15): p. 5715-27, doi:10.1128/MCB.00001-06.
100. Ye, J., et al., *Primer-BLAST: a tool to design target-specific primers for polymerase chain reaction.* BMC Bioinformatics, 2012. **13**: p. 134, doi:10.1186/1471-2105-13-134.
101. Bribian, A., et al., *Role of the cellular prion protein in oligodendrocyte precursor cell proliferation and differentiation in the developing and adult mouse CNS.* PLoS One, 2012. **7**(4): p. e33872, doi:10.1371/journal.pone.0033872.
102. Delisle, J.C., et al., *Bipotential mouse embryonic liver (BMEL) cells spontaneously express Pdx1 and Ngn3 but do not undergo further pancreatic differentiation upon Hes1 down-regulation.* BMC Res Notes, 2008. **1**: p. 136, doi:10.1186/1756-0500-1-136.
103. Shawki, H.H., et al., *MAFB is dispensable for the fetal testis morphogenesis and the maintenance of spermatogenesis in adult mice.* PLoS One, 2018. **13**(1): p. e0190800, doi:10.1371/journal.pone.0190800.
104. Ip, W., et al., The Wnt signaling pathway effector TCF7L2 is upregulated by insulin and represses hepatic gluconeogenesis. Am J Physiol Endocrinol Metab, 2012. 303(9): p. E1166-76, doi:10.1152/ajpendo.00249.2012.
